# Flexible and multifaceted: the plasticity of renin-expressing cells

**DOI:** 10.1007/s00424-022-02694-8

**Published:** 2022-05-05

**Authors:** Katharina A. E. Broeker, Julia Schrankl, Michaela A. A. Fuchs, Armin Kurtz

**Affiliations:** Institute of Physiology, University of Regensburg, Universitätsstraβe 31, D-93053 Regensburg, Germany

**Keywords:** Renin, Renin–angiotensin–aldosterone system, Erythropoietin, Phenotypic transformation, Renal interstitial cells, Regeneration

## Abstract

The protease renin, the key enzyme of the renin–angiotensin–aldosterone system, is mainly produced and secreted by juxtaglomerular cells in the kidney, which are located in the walls of the afferent arterioles at their entrance into the glomeruli. When the body’s demand for renin rises, the renin production capacity of the kidneys commonly increases by induction of renin expression in vascular smooth muscle cells and in extraglomerular mesangial cells. These cells undergo a reversible metaplastic cellular transformation in order to produce renin. Juxtaglomerular cells of the renin lineage have also been described to migrate into the glomerulus and differentiate into podocytes, epithelial cells or mesangial cells to restore damaged cells in states of glomerular disease. More recently, it could be shown that renin cells can also undergo an endocrine and metaplastic switch to erythropoietin-producing cells. This review aims to describe the high degree of plasticity of renin-producing cells of the kidneys and to analyze the underlying mechanisms.

## Introduction

The kidneys are the main production site for renin, the key enzyme of the systemic renin–angiotensin–aldosterone system (RAAS). The systemic RAAS plays an essential role in regulating blood pressure and maintaining electrolyte and extracellular volume homeostasis. The protease renin is the limiting determinant of the RAAS by cleaving the decapeptide angiotensin (Ang) I from angiotensinogen which is mainly produced in the liver. Endothelial cell-derived angiotensin-converting enzyme (ACE) shortens Ang I to Ang II that exerts its effects mainly via Ang II type I (AT_1_) receptors. Ang II has a strong vasoconstrictor effect and thus directly influences blood pressure. Additionally, it regulates the production of aldosterone in the adrenal cortex and the release of antidiuretic hormone from the pituitary gland, thereby ensuring salt and water homeostasis [104, 106].

Renin production and secretion are mainly controlled by three different mechanisms. These are the renal perfusion pressure, the tubular sodium chloride concentration sensed by macula densa cells, and the activity of β-adrenergic receptors on renin-producing cells. More generally renin production and secretion are controlled in the sense of negative feedback loops involving blood pressure, sodium balance, and angiotensin II [9, 96].

Most of the circulating renin is produced by specialized myoendocrine cells located in the walls of afferent arterioles directly at the entrance into the glomerulus. Due to this characteristic position in the adult, renal renin-producing cells are called juxtaglomerular cells. When the demand for renin increases due to threats to sodium or blood pressure homeostasis, additional cells in the afferent vessel wall and in the juxtaglomerular area are recruited to adjust the production of renin accordingly. Once homeostasis is restored, renin production ceases in recruited cells which retransform into their former phenotype [27, 100, 106]. This regulatory mechanism hints at a high plasticity of renal renin-producing cells.

Plasticity is not only relevant for the physiological regulation of renin production. Renin-lineage cells can also fulfill a kind of stem cell function as progenitor cells for the regeneration of mesangial cells and podocytes after kidney injury [43, 86, 105]. Moreover, it was found that juxtaglomerular renin cells can transform into erythropoietin (EPO)-producing cells due to genetic activation of the hypoxia signaling pathway [6, 48].

**Fig. 1:**
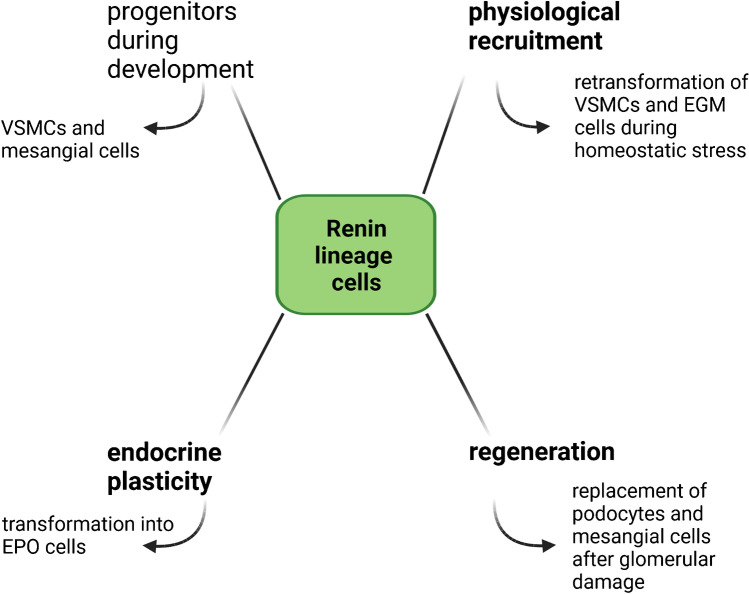
Schematic overview of different aspects of renin-lineage cell plasticity. Renin-lineage cells show a high plasticity and can fulfill different functions in order to maintain renal function, blood pressure, and water and salt balance in the body. Topics highlighted in this review are indicated in bold font

This review will specifically focus on the aspect of renin-cell plasticity (Fig. 1). The identity of classic juxtaglomerular renin^+^ cells and the newly described interstitial renin-expressing cells will be outlined in more detail. Particular attention will be paid to processes involved in the reversible metaplastic transformation of cells into renin-producing cells. Another focus will be the involvement of renin-lineage cells in regeneration and the endocrine transformation of renin^+^ cells into EPO-producing cells.

## Identity of renin-expressing cells

### “Classic” renin cells at juxtaglomerular position

The classic renin-producing cells are located directly at the entrance of the afferent arteriole into the glomerulus, hence their labeling as juxtaglomerular cells. Together with the adjacent parts of the afferent and efferent arteriole, the extraglomerular mesangium (EGM) and the specialized macula densa cells of the distal tubule, they form the juxtaglomerular apparatus (JGA)—a functional unit, which coordinates the secretion rate of renin, the glomerular filtration rate and the tubuloglomerular feedback. Thus, it ultimately regulates systemic blood pressure as well as salt and extracellular volume balance, by integrating various signals.

In the adult mammal, only about 4–8 renin-producing cells per glomerulus can be detected under basal physiologic conditions [106]. The juxtaglomerular renin-producing cells originate in the FoxD1^+^ stromal progenitor cell population which also gives rise to interstitial fibroblasts and pericytes, mesangial cells, and vascular smooth muscle cells (VSMCs) [21, 37, 101]. Renin-producing cells can first be detected sporadically in the undifferentiated mesenchyme. With the onset of kidney vascularization, renin-producing cells are involved in the development of the renal vascular tree. Before a new arterial branch grows out, renin cells accumulate at the branching point. During outgrowth and elongation of the vessel, the renin cells constitute the mural cells of the newly developing vascular segments. With ongoing vessel maturation, the renin-producing cells differentiate into VSMCs. Thus, the renin^+^ cells are always associated with the youngest vessel segments. With the completion of the vascular tree, they are restricted to the juxtaglomerular position. Genetic defects in the RAAS cascade or deletion of the renin gene during nephrogenesis lead to severe vascular malformations [10, 24, 44, 92, 102]. In adults, the renin-lineage cells still retain their developmental plasticity and retransform into the renin cell phenotype when homeostasis is threatened [27, 100].

Renin-producing cells are located in the media layer of the afferent arterioles and have a cuboidal shape. The cells are packed with storage vesicles both small individual vesicles and also voluminous interconnected caverns [107]. Renin is synthesized as the preproprotein prorenin, which is glycosylated in the endoplasmic reticulum and then proteolytically processed to active renin in the storage vesicles [71], from which it is released by compound exocytosis on demand [107]. In addition, prorenin is constitutively secreted. Upon binding to the (pro)renin receptor, prorenin can mediate either RAS-dependent or RAS-independent effects [77]. The cells forming the juxtaglomerular apparatus are interconnected by numerous gap junctions. Within the JGA, gap junctions are formed by four different connexin (Cx) isoforms, Cx37, Cx40, Cx43, and Cx45, with renin-producing cells expressing only Cx37 and Cx40 [49, 50, 118]. Expression of Cx40 seems to be crucial for determining the juxtaglomerular position of renin-producing cells, as well as for the blood pressure-dependent regulation of renin synthesis and secretion [47, 51, 61, 112, 113]. Inducible deletion of Cx40 in adult mice results in misplaced renin cells that are no longer located in the media layer of afferent arterioles but in the extraglomerular mesangium [22]. In addition, the inhibitory effect of elevated blood pressure on renin synthesis and secretion is absent, resulting in increased plasma renin concentrations despite hypertension [22, 113]. Thus, Cx40 apparently mediates the pressure control of renin secretion [61]. A study with mice harboring a missense mutation in the Cx40 gene, first observed in a human patient displaying hypertension, suggests that correct Cx40 gap junction coupling of renin-producing cells is also essential for renin secretion in the human kidney [58]. A study, in which Cx40 was replaced by Cx45, however shows that not the expression of Cx40 itself is crucial for the regulation of renin release from juxtaglomerular cells but the gap junctional coupling with neighboring cells [98]. Moreover, not only cell–cell communication via gap junctions seems to be important for the regulation of renin secretion but also signaling via connexin hemichannels [36]. A recent study suggests that pannexin 1 could also be involved in the regulation of basal renin secretion through mediating intercellular communication [14]. Another cell adhesion molecule essential for mediating cell–cell or cell–matrix interactions is β1-integrin, which is abundantly expressed in the kidneys including renin-lineage cells. Deletion of β1-integrin in the renin-lineage results in apoptosis and therefore leads to a decrease in circulating renin, hypotension, and vascular alterations in the kidney [67].

To this date, a number of molecular markers have been determined that are characteristically expressed in juxtaglomerular renin-producing cells. They strongly express the AT_1_ receptor that mediates the effects of Ang II [95]. Moreover, they express aldo–keto reductase 1b7 (Akr1b7), an enzyme that reduces carbonyl groups to their respective alcohols [54, 57]. Prolyl-4-hydroxylase (PHD) 3, an enzyme involved in the hypoxia signaling pathway, is also expressed in renin-producing cells [6]. Cell type-specific deletion of the respective marker, however, has no direct effect on renin secretion [6, 60, 76].

Through numerous studies in mice two major signaling pathways have emerged that play a key role in determining and maintaining the renin cell phenotype. One is the Notch signaling pathway and the other is the cAMP signaling pathway. Notch signaling is a cell–cell communication pathway involved in regulating cell fate and thereby enabling cell plasticity. Ligand binding to the transmembrane Notch receptor leads to cleavage of the extracellular receptor domain followed by a second cleavage step that releases the intracellular domain of Notch (NICD). NICD translocates into the nucleus and binds to the recombination signal binding protein for Ig-kJ region (RBP-J), an important downstream effector of Notch signaling. The DNA-binding protein RBP-J recruits a coactivator complex and thereby activates the transcription of Notch target genes [4, 23]. In juxtaglomerular cells, RBP-J activates genes that are characteristic for their myo-endocrine phenotype and simultaneously inhibits the expression of genes typical for other cell lineages like hematopoietic markers. After deletion of RBP-J, renin-lineage cells stop producing renin, lose the typical renin vesicles, and no longer express Akr1b7 or contractile proteins [7, 8]. Another factor critically involved in Notch signaling and renin cell identity is the enzyme Dicer which is responsible for microRNA-processing [18, 39, 103]. miRNAs regulate many cellular processes by targeting specific mRNAs to either suppress or activate their expression [17, 81]. For the identity of renin cells, two distinct miRNAs, miR-330 and miR-125b-5p, seem to be important [68].

The second major pathway which is not only crucial for the synthesis and secretion of renin but also for the differentiation and maintenance of renin-producing cells is cAMP signaling. cAMP activates protein kinase A, which subsequently leads to the phosphorylation of cAMP-responsive element binding protein (CREB) in the nucleus. Phosphorylated CREB as a complex with the co-factors CBP (CREB-binding protein) and p300 then binds to the cAMP-responsive element in the promoter region of target genes like renin and Akr1b7 [54, 99]. A rise in intracellular cAMP to stimulate renin expression can be mediated either by the G_s_/AC-coupled prostaglandin E_2_ receptors EP_2_ and EP_4_ or G_s_/AC-coupled β-adrenergic receptors (G_s_/AC, G protein stimulating cAMP signaling by adenylyl cyclase (AC) activation) [9, 97]. Deletion of either β-adrenergic receptors, both EP_2_ and EP_4_ receptors or the intracellular G_sα_ (G protein subunit α) results in significantly decreased renin expression and secretion in adult mice [11, 15, 16, 45]. Also, combined deletion of the two histone acetyltransferases CBP and p300 in renin-lineage cells results in a loss of renin-expressing cells [26].

These findings are further supported by more recent studies revealing the molecular fingerprint of renin-producing cells through analysis of the chromatin structure and epigenetic status of juxtaglomerular cells, recruited renin cells, and the renin cell line As4.1. Renin-lineage cells possess a unique super-enhancer at the renin gene locus which directly influences renin expression. In addition, they share 91 super-enhancers that determine the renin cell phenotype. More in-depth data analyses further confirmed the importance of Notch and cAMP signaling for determining renin cell identity [64]. In this context, it was shown that the 3D structure of chromatin and, in particular, chromatin structural protein Ctcf (CCCTC-binding factor) are also crucial for the expression of renin and the number of renin-producing cells [63].

### Interstitial renin-expressing cells

Recently, two groups independently reported active renin gene transcription in tubulointerstitial cells of the adult mouse kidney [6, 70]. Renal interstitial fibroblast-like cells are involved in maintaining tissue architecture and integrity by producing extracellular matrix and mediating the cross-talk between different renal cell types. In many kidney diseases, interstitial fibroblasts and pericytes play a crucial role in the development of interstitial fibrosis as matrix-producing myofibroblasts [37]. Moreover, in healthy kidneys, a subset of interstitial fibroblasts produce the hormone erythropoietin (EPO), which regulates erythropoiesis [1, 67]. Like juxtaglomerular renin-producing cells, renal interstitial fibroblasts/pericytes originate from the FoxD1^+^ stromal progenitor cell population [101].

**Fig. 2:**
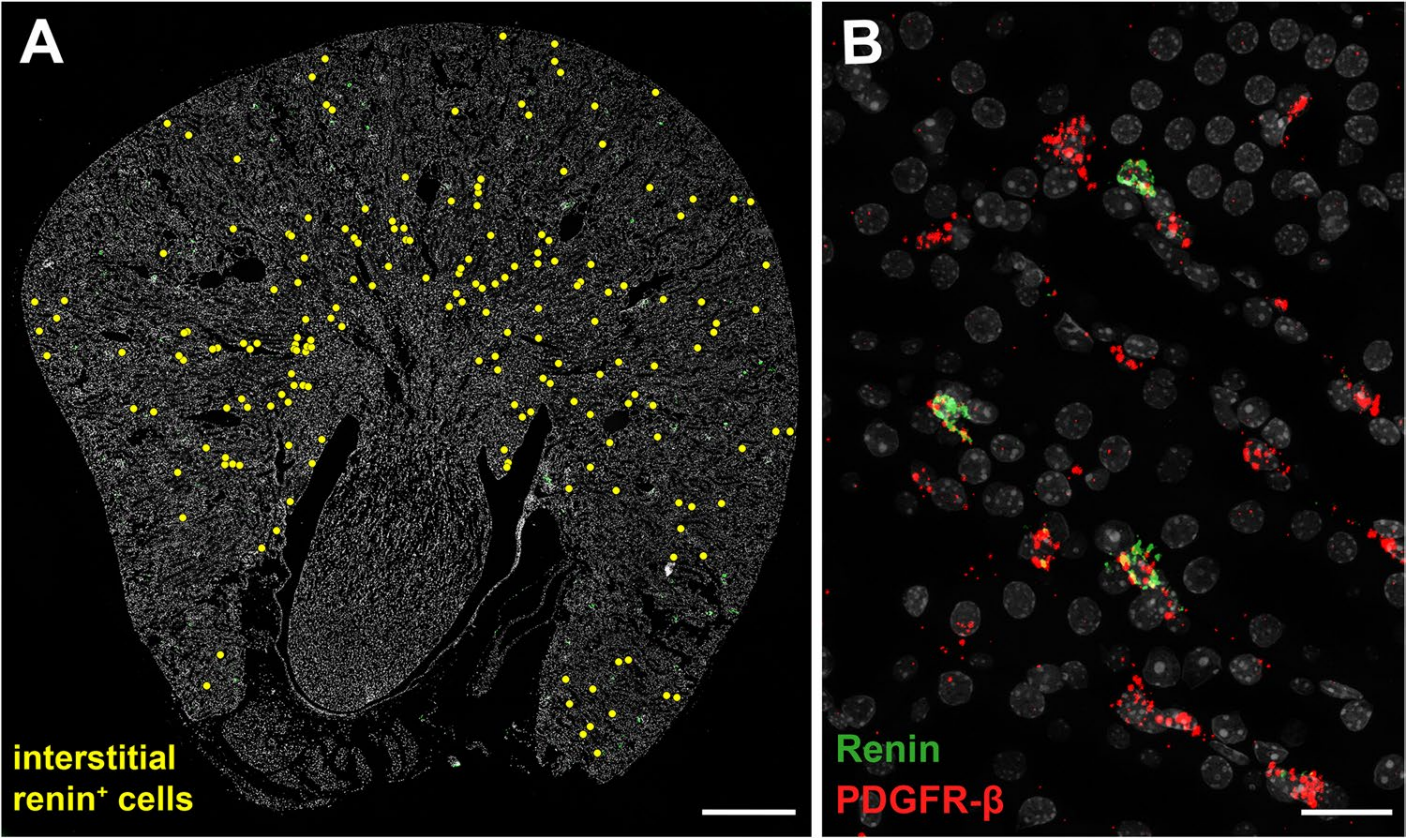
Interstitial renin-expressing cells on kidney sections of wild-type mice. **A** Spatial distribution pattern of interstitial renin-expressing cells was visualized with RNAscope, a high resolution in situ hybridization technology. Renin mRNA-expressing cells were highlighted with yellow dots on a kidney section of a wild-type mouse. Interstitial renin mRNA-expressing cells are mainly distributed in the outer medulla and to a lesser extent in the renal cortex. Nuclei were counterstained with DAPI (gray). Scale bar 500 μm. **B** Medullary detail of a co-RNAscope for renin (green) and PDGFR-β (red) mRNA on a wild-type kidney section. Colocalization of both mRNAs identifies interstitial renin^+^ cells as fibroblast-like cells. Nuclei were counterstained with DAPI (gray). Scale bar 20 μm

Under basal conditions, about 250 interstitial cells expressing renin mRNA per transverse kidney section of wild-type mice (C57/Bl6J) can be detected using the high-resolution RNAscope in situ hybridization technique. Most of these interstitial renin-expressing cells are found throughout the outer medulla and some are dispersed throughout the tubulointerstitium of the cortex. These interstitial renin-expressing cells coexpress the fibroblast marker platelet-derived growth factor receptor β (PDGFR-β) (Fig. 2) [6, 70] which is also expressed by EPO-producing fibroblasts [20]. Within the pool of PDGFR-β^+^ interstitial cells, a number of different subpopulations exists that are characterized by the expression of a distinct additional marker like CD73, Gli1, smooth-muscle myosin heavy chain (SM-MHC), or tenascin-C [5]. Whether interstitial renin expression is restricted to one of these subpopulations has not yet been determined. Like typical renal fibroblasts, interstitial renin^+^ cells possess a flat, elongated cell body with processes which often wrap around tubules or capillaries [52]. In contrast to juxtaglomerular cells, interstitial renin-expressing cells are negative for renin immunohistochemistry in the kidneys of wild-type mice under basal conditions. However, lineage tracing using a GFP reporter mouse confirmed the activity of the renin gene promoter [6]. Therefore, it could be speculated that interstitial renin-expressing cells do not store renin in granules but instead constitutively release prorenin [71]. Whether the interstitial renin^+^ cells also possess the typical markers of juxtaglomerular renin-producing cells, like Akr1b7, AT_1A_, Cx40 or distinct miRNAs requires further investigation.

So far, the functions of renin expression in interstitial cells under physiological or pathophysiological conditions are not understood. An initial study suggests that hypotension caused by anemia or treatment with an angiotensin II receptor blocker increases the number of interstitial renin-expressing cells, whereas hypertension decreases their number compared to control animals. Additional interstitial renin^+^ cells are recruited throughout the cortex and outer medulla. In this context, it was speculated that interstitial renin expression may contribute to systemic renin production to maintain blood pressure homeostasis. Notably, some interstitial renin^+^ cells coexpress EPO in the anemic hypotension mouse model (please see part IV for detailed discussion). Furthermore, an increased number of interstitial renin-expressing myofibroblasts is observed in an experimental fibrosis model [70]. However, in a mouse model of aldosterone synthase deficiency leading to hypotension and significantly increased plasma renin concentrations, the number of interstitial renin^+^ cells is unchanged compared to wild-type animals [6, 62]. The increased levels of circulating renin in these mice are produced mainly by recruited cells along the afferent arterioles, in the extraglomerular mesangium, and in perivascular cells [62]. Therefore, it is conceivable that juxtaglomerular renin production and interstitial renin expression are two separate systems that are regulated independently. While juxtaglomerular renin regulates the systemic RAAS, interstitial renin expression may belong to a local RAS, which apart from the kidney has also been described in the heart, brain, or the adrenal glands [82, 117]. The intrarenal RAS is a local paracrine system, which contains all elements required for Ang II production and is involved in the pathogenesis of hypertension and renal disease. In diabetic nephropathy or Ang II-induced hypertension, for example, components of the RAS like angiotensinogen, ACE, (pro)renin, and AT_1_ receptors are upregulated along the nephron and in the medullary region leading to high levels of intratubular Ang II [28, 73, 117]. However, the detailed functions of the intrarenal RAS and its regulation are not yet fully understood.

## Cells reversibly recruited for renin production

### Vascular smooth muscle cells in afferent arterioles

If a homeostatic threat to blood pressure or sodium balance develops that cannot be compensated by increasing the secretion rate of the existing juxtaglomerular renin-producing cells, additional cells are recruited for renin production rather than renin synthesis per cell is upregulated. This is the case, for example in states of hypotension, salt depletion, or restrictions of extracellular volume which ultimately lead to a reduction of renal blood flow [3, 25, 29]. The recruitment of additional renin-producing cells does not occur by proliferation of the existing juxtaglomerular renin cells, but largely by retransformation of cells that had already expressed renin during renal development and then differentiated into other cell types after completion of kidney development [31]. Depending on the strength of the stimulus, initially vascular smooth muscle cells along the afferent arterioles are recruited for renin synthesis. If the stimulus persists for a longer period of time, subsequently more and more cells are recruited also along larger vessels and in the intra- and extraglomerular mesangium [100]. Recently, evidence was provided suggesting that a small proportion (≤ 10%) of renin-producing cells in the adult mouse also arise by neogenesis, that is, de novo differentiation from other cells [35]. Once homeostasis is restored, the recruited cells retransform back to their previous phenotype [27].

**Fig. 3:**
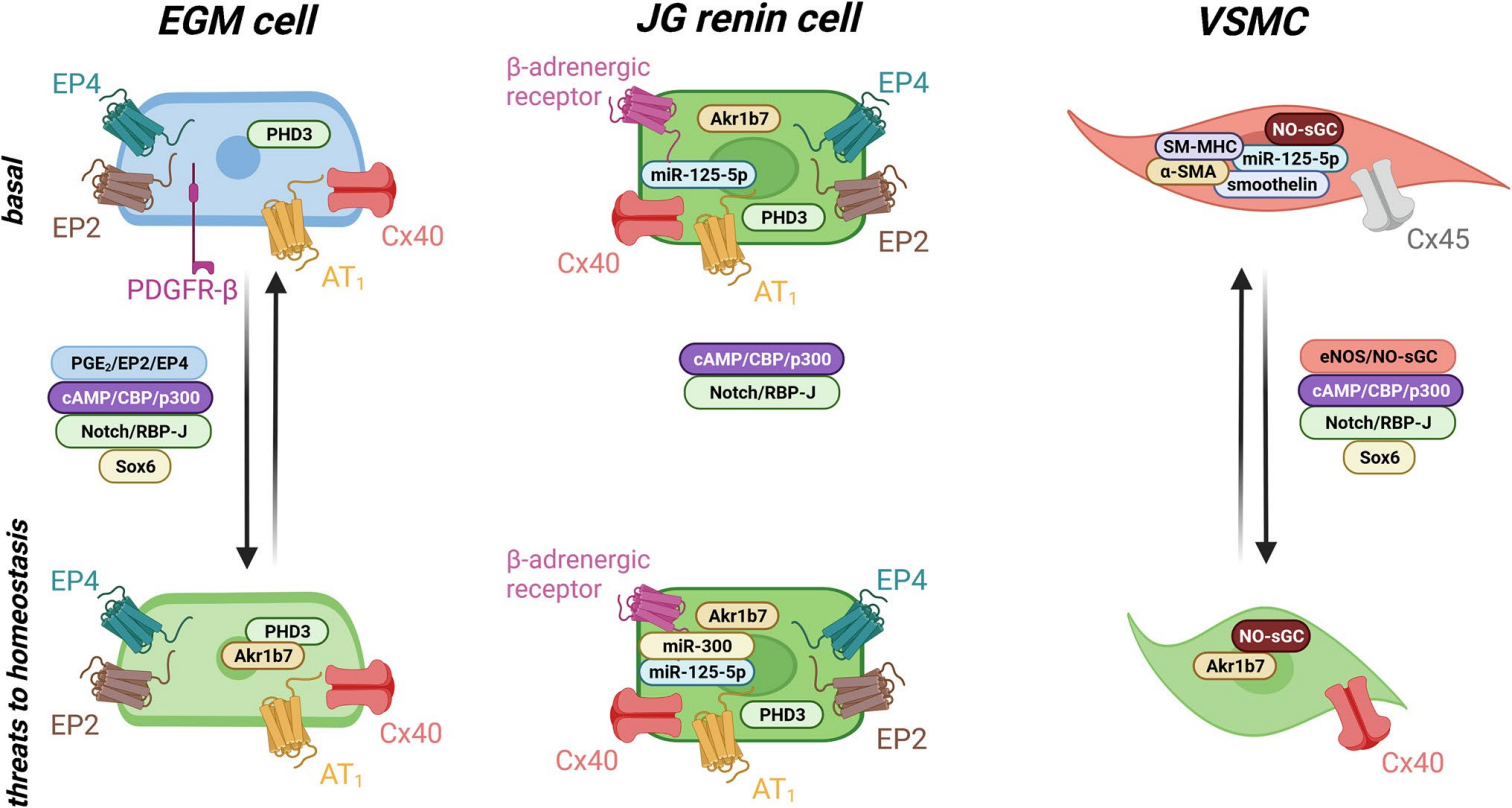
Schematic overview of distinct markers conveying the identity of juxtaglomerular renin cells and of signaling pathways involved in recruitment of extraglomerular (EGM) cells and vascular smooth muscle cells (VSMCs) for renin production. Phenotypic changes accompanying recruitment are also depicted

In general, VSMCs are crucial for the control of blood pressure and blood distribution as well as for maintaining the blood vessel integrity. Due to their contractile function, they express a distinct set of contractile proteins including α-SMA (smooth muscle α-actin), SM22α, SM-MHC (smooth-muscle myosin heavy chain), smoothelin, and calponin [56]. Contractile VSMCs show an elongated spindle-like shape and possess a large number of contractile filaments [89]. Once VSMCs are recruited for renin synthesis, they become more epitheloid. They acquire renin storage granules and the endoplasmic reticulum as well as the Golgi apparatus are more pronounced, whereas the number of myofibrils decreases [27]. Along with renin, the recruited VSMCs begin to express typical renin cell markers, such as Akr1b7 and Cx40. In contrast, VSMC marker expression is downregulated. Translation of typical proteins such as Cx45, α-SMA, SM-MHC, and smoothelin can no longer be observed in these cells (Fig. 3) [42, 47, 84].

Prerequisite for the transformation of a cell into a renin-producing cell seems to be the ingrained epigenetic memory of the renin cell phenotype. This molecular memory resides in a set of 91 super-enhancers common to renin-lineage cells and an additional super-enhancer located at the renin gene locus. This super-enhancer seems to directly control renin gene expression and to integrate various signals regarding blood pressure and salt and volume homeostasis [64]. Sox6 is a transcription factor that appears to be important in this context. Sox6 binds to the renin promoter within the super-enhancer region. After deletion of Sox6 in renin-lineage cells, recruitment of VSMCs is impaired under salt restriction and dehydration or in renal arterial stenosis [90, 91]. Also, posttranscriptional modifications play an important role in retransformation. Deletion of the RNase III endonuclease Dicer, an enzyme necessary for the maturation of miRNAs, leads to a decreased number of renin-producing cells [103]. Two distinct miRNAs are involved in balancing the myoendocrine phenotype of renin-producing cells. Usually, all renin-lineage cells express miR-125-5p which maintains their contractile functions. When homeostasis is threatened, the expression of miR-125-5p decreases in VSMCs in favor of the endocrine phenotype. In juxtaglomerular renin cells, however, miR-125-5p expression is maintained. In addition, they start to express miR-330 which enhances their endocrine features [68].

Moreover, Notch/RBP-J signaling and the cAMP/CBP/p300 pathway that are involved in determining the renin cell phenotype are also important for the retransformation of VSMCs into renin-producing cells. RBP-J acts as a transcription factor thereby regulating not only genes that confer the endocrine phenotype (renin and Akr1b7), but also the contractile phenotype (α-SMA, SM-MHC, and smoothelin). Thus, RBP-J activity is essential for the recruitment of VSMCs during homeostatic challenges. Mice with deletion of RBP-J in renin-lineage cells were unable to recruit VSMCs when treated with the ACE inhibitor captopril in combination with a low sodium diet [8].

Activation of the cAMP/CBP/p300 pathway contributes to the transformation of VSMCs into renin-producing cells [83]. In contrast to juxtaglomerular renin cells, cAMP in VSMCs does not seem to be generated by activation of EP_2_/EP_4_ receptors or β-adrenergic receptors [42]. Deletion of β-adrenergic receptors in renin-lineage cells does not impair the recruitment of VSMCs in mice treated with a low sodium diet and an ACE inhibitor [75]. Intracellular cAMP can also be increased by inhibiting phosphodiesterase 3. This inhibition can be mediated by increased cGMP levels, generated through the activation of the NO-sensitive soluble guanylate cyclase (sGC) [65]. This NO-sGC-mediated pathway seems to be of relevance for the transformation of VSMCs. Deletion of either endothelial NO-synthase (eNOS) or sGC leads to an impaired recruitment of VSMCs in mice on a low sodium diet in combination with an ACE inhibitor. But, the recruitment of EGM cells is unaffected in these mouse models (Fig. 3) [74].

Homeostatic threats lead to recruitment of VSMCs and EGM cells for additional renin production. The transformation of EGM cells depends on the activation of prostaglandin receptors EP_2_ and EP_4_ and subsequently cAMP/CBP/p300 signaling as well as Notch signaling. Transformed EGM cells lose PDGFR-β and instead express Akr1b7. VSMCs are recruited by the activation of cAMP/CBP/p300 signaling through NO-sGC and Notch signaling. The recruited VSMCs lose their contractile features and become more cuboid in their appearance. Moreover, they start to express Cx40 instead of Cx45.

### Extraglomerular mesangial cells

Extraglomerular mesangial cells are a part of the juxtaglomerular apparatus. Like renin-producing cells, they express Cx40, AT_1_, and the prostaglandin E_2_ (PGE_2_) receptors EP_2_ and EP_4_ [16, 95]. Moreover, they are positive for PDGFR-β, the common marker of renal interstitial cells and pericytes. Transformation of extraglomerular mesangial cells to renin-producing cells is often observed in situations of chronic salt wasting, for example, due to genetic defects in the aldosterone synthase or in chloride channels, as in Bartter syndrome [2, 30, 62, 108]. After transformation into renin-producers, EGM cells start to express Akr1b7 [54]. PDGFR-β expression however is downregulated (Fig. 3) [41].

Renin production by EGM cells seems to be preferentially regulated through the activation of EP_2_ and EP_4_ receptors. These G_s_-coupled receptors are activated through binding of PGE_2_ which is produced by cyclooxygenase 2 (Cox-2) in the macula densa cells. PGE_2_ production is upregulated by salt deficiency. Activation of EP_2_ and EP_4_ receptors leads to an increase in the intracellular cAMP levels, thereby elevating renin expression [9]. Increased plasma renin levels in humans on a low salt diet could be suppressed by the administration of selective Cox-2 inhibitors [40]. The importance of the Cox-2/PGE_2_/EP axis for renin expression is further supported by the successful treatment of hyperreninemia with Cox-2 inhibitors in patients with Bartter syndrome [80, 88]. Deletion of both EP_2_ and EP_4_ receptors or treatment with a Cox-2 inhibitor in mice lacking aldosterone synthase significantly downregulates extraglomerular renin expression but renin production by VSMCs is not affected [42]. The persistent renin expression by VSMCs in these mice is supported by a recent study reporting a lack of EP_2_ and EP_4_ receptors in these cells [16]. In contrast, signaling via NO-sGC, which is important for VSMC recruitment, has no effect on EGM recruitment (Fig. 3) [74].

In unstressed wild-type mice, the expression of Cx40 seems to suppress the expression of renin in EGM cells. Deletion of Cx40 leads to renin expression in extraglomerular mesangial cells, while the typical juxtaglomerular renin expression is absent [22]. However, recruitment of VSMCs for renin production seems unaffected in Cx40-deficient mice [61].

## Renin cell plasticity in pathophysiology

Besides the physiological recruitment, renin-lineage cells have been suggested to play a role in the regeneration of different glomerular cell types after injury. In a model of focal segmental glomerular sclerosis, tracking of renin-lineage cells with reporter mice demonstrated that inhibition of the RAS by administration of the ACE inhibitor enalapril or the AT_1_ receptor inhibitor losartan stimulated the proliferation of renin-lineage cells in the juxtaglomerular region. These cells then migrated into the glomerulus and differentiated into intraglomerular mesangial cells, podocytes, or epithelial cells of Bowman’s capsule. After transdifferentiation, the cells no longer express renin, but instead express typical markers of the respective cell types. The mesangial cells differentiated from the renin-lineage cells are positive for integrin α8, the podocytes express synaptopodin, nephrin, podocin, and Wilms tumor protein, and the parietal epithelial cells are positive for PAX8 and claudin [43, 53, 86]. These findings are supported by another study, where after induction of renal damage and a loss of podocytes due to 5/6 nephrectomy, renin-lineage cells also migrated into the glomeruli and transformed into podocytes [85]. These findings may explain why therapies with RAS inhibitors (RASi) have been shown to be beneficial in patients with glomerular disease [32, 59, 115]. The signaling pathways that induce the migration and the phenotypic shift of renin-lineage cells into podocytes and parietal epithelial cells are not yet known [53].

In another model of mesangial cell injury, extraglomerular renin-lineage cells could also compensate for the loss of intraglomerular mesangial cells through migration and retransformation. It has been speculated that pathways which mediate the differentiation of renin-lineage cells during nephrogenesis could be involved in this repair process [105].

A quite recent study showed that prolonged treatment with RASi activates renin-producing cells and leads to arterial hypertrophy, a concentric thickening of the intrarenal arteries and arterioles in mice and humans. The renin^+^ cells adapt a more VSMC-like phenotype with upregulated expression of α-SMA, SM-MHC, and calponin-1, while maintaining renin expression. Based on these findings, further studies are needed to determine more precisely the morphologic and functional consequences of RASi treatment to be able to balance its positive and negative effects in the future [72, 114].

In rare cases, transformation of juxtaglomerular renin cells into tumor cells occurs. Juxtaglomerular cell tumors are benign and are also referred to as reninomas. They secrete renin autonomously and persistently, leading to increased plasma renin activities and elevated serum aldosterone levels. Affected patients therefore suffer from hypertension, hyperreninemia, and secondary aldosteronism [55, 109]. Findings suggest that impaired expression of RBP-J could lead to overexpression of renin in juxtaglomerular cell tumors [38]. After transformation reninoma cells newly express CD34, a marker for hematopoietic stem cells and collagen type VI [55, 66]. To this date, the signals and regulatory mechanisms involved in the regenerative or aberrant transformation await elucidation.

## Endocrine plasticity: transformation into erythropoietin producers

Recently, it has been shown that a subset of interstitial fibroblast-like cells expresses renin in mouse kidneys. In situations of stimulated renal erythropoietin (EPO) production, a partial coexpression of renin and EPO in interstitial cells has been observed [6, 70].

Apart from renin, the kidneys are also the main expression site for the hormone EPO which triggers erythropoiesis. EPO is produced by tubulointerstitial fibroblasts located in the deep cortex along the cortico-medullary border. These cells are characterized by the expression of PDGFR-β and CD73. EPO expression is stimulated by a fall of the local oxygen tension as a consequence of arterial hypoxia or anemia. EPO production is transcriptionally controlled by the dimeric transcription factor HIF-2. Under normoxic conditions, the HIF-2α subunit is continuously hydroxylated by prolyl-4-hydroxylase 2 (PHD-2). Hydroxylated HIF-2α gets ubiquitinylated by the ubiquitin E3 ligase von Hippel-Lindau (Vhl) protein and eliminated by proteasomal degradation. If tissue oxygenation declines, HIF-2α is no longer hydroxylated and degraded but instead translocates into the nucleus. There, HIF-2α dimerizes with the HIF-1β subunit and together with other co-factors, and activates the transcription of its target genes, including EPO [33, 79].

**Fig. 4:**
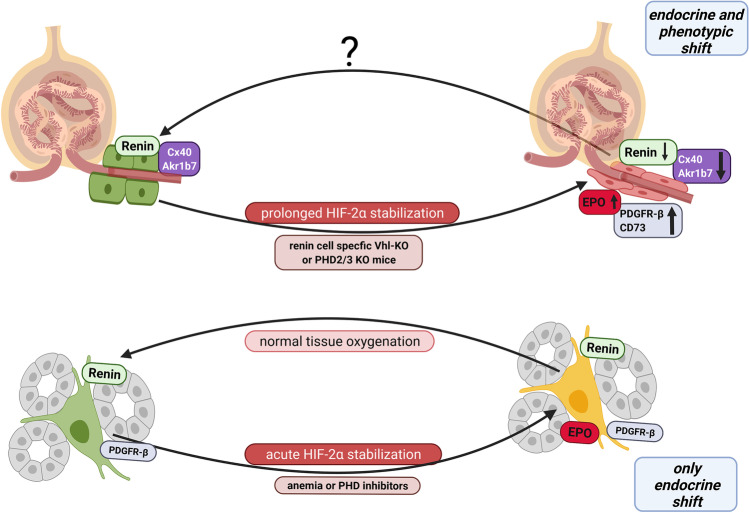
Schematic overview illustrating the transformation of juxtaglomerular or interstitial renin^+^ cells into EPO-producing cells

It has been observed in mice that during anemia or after pharmacological inhibition of PHDs a subpopulation of interstitial cells coexpresses EPO and renin suggesting an endocrine plasticity of these cells [70]. In addition, renin cell-specific deletion of PHD2 also induces EPO expression in interstitial cells [79], indicating that interstitial renin^+^ cells are able to express EPO. Altogether, these findings suggest that at least a subpopulation of interstitial renin^+^ cells rapidly turns on EPO expression in response to an acute stimulus that leads to HIF-2α stabilization [6]. In contrast to the transformation of VSMCs for renin production, no phenotypic shift can be observed after the induction of EPO expression in interstitial renin^+^ cells. The renin and EPO coexpressing interstitial cells still express the typical fibroblast marker PDGFR-β (Fig. 4) [6].

In contrast to interstitial renin^+^ cells, EPO expression is not induced in juxtaglomerular renin-producing cells after treatment with a single-dose of a PHD inhibitor or after renin cell-specific deletion of PHD2 [6]. At first glance, these findings appear contradictory to the original observation that renin cell-specific deletion of Vhl induces EPO expression in juxtaglomerular cells [19, 48]. It turned out, that it requires a codeletion of PHD2 with PHD3 to induce EPO expression in juxtaglomerular renin cells [6]. PHD3 is a PHD isoform known to cooperate with PHD2 in some cell types to modulate the HIF response [69, 111]. Clear HIF-2α stabilization is observed in juxtaglomerular renin cells with Vhl or with PHD2/PHD3 deletions, while deletion of PHD2 alone, only causes a minor HIF-2α stabilization. Although single-dose application of PHD inhibitors leads to HIF-2α stabilization in juxtaglomerular cells, it fails to induce EPO gene expression in these cells [6, 46]. These findings suggest that EPO expression in juxtaglomerular renin cells requires prolonged HIF-2α stabilization. This could indicate that a metaplastic cell transformation is a prerequisite for the inducibility of EPO expression in juxtaglomerular cells.

After Vhl-deletion or PHD2/3 codeletion juxtaglomerular renin^+^ cells lose their renin storage granules and no longer appear epithelioid. Instead, they have a rather flattened and elongated appearance similar to fibroblast-like cells. Moreover, not only the expression of renin but also the expressions of the typical markers Akr1b7 and Cx40 are strongly downregulated. Conversely, typical markers of interstitial EPO-producing cells such as PDGFR-β and CD73 are clearly upregulated in transformed juxtaglomerular cells (Fig. 4) [6, 19, 46]. Whether this metaplastic transformation of juxtaglomerular renin^+^ cells into EPO^+^ cells is reversible, like for recruited VSMCs, still needs to be determined.

Juxtaglomerular renin cells (top, left), expressing Cx40 and Akr1b7, are transformed into EPO-producing cells by a prolonged genetic stabilization of HIF-2α. In juxtaglomerular renin cells, HIF-2α leads to a phenotypic as well as an endocrine shift. After transformation, these cells no longer have their distinct cuboid shape, but appear flattened, and instead of Cx40 and Akr1b7, these cells then express PDGFR-β and CD73 (top, right). Whether a re-transformation of EPO cells in juxtaglomerular position, back into renin-producing cells is possible, is not yet known.

Interstitial renin-expressing cells (bottom, left) can undergo an endocrine shift in response to an acute stabilization of HIF-2α due to anemia or the administration of PHD inhibitors. Interstitial renin-expressing cells are able to coexpress renin and EPO simultaneously (bottom right). If the acute hypoxic stimulus is removed, interstitial EPO/renin^+^ cells revert to only renin expression.

The mechanisms underlying the transformation of juxtaglomerular cells in EPO-producing cells are still unknown. So far, it is only known that the metaplastic transformation of renin^+^ cells into EPO-producing cells is dependent on HIF-2α and can be prevented by the deletion of HIF-2α in these cells. However, the pathways interacting with the hypoxia signaling pathway to mediate this transformation are still elusive [19, 46]. It is conceivable that activation of the hypoxia signaling pathway via HIF-2 leads to a change in the microRNA expression pattern [48, 63]. miRNAs can directly affect chromatin structure by targeting factors like DNA methyltransferases or histone deacetylases [4]. Previous studies have already demonstrated that the chromatin structure and the expression of certain miRNAs are essential for renin cell identity, as well as for the recruitment of VSMCs for renin production [67, 68, 101]. If the expression of miRNAs changes, this could lead to a remodeling of chromatin structure and thus ultimately influence cell identity.

It can also be speculated that HIF-2α activation could lead to a de-differentiation of juxtaglomerular renin^+^ cells to a less specialized fibroblast-like cell. It was shown in tumor cells that chronic HIF-2 stabilization can lead to a phenotype shift of differentiated cells towards a more de-differentiated phenotype. For example, neuroblastoma cells lose typical neuroendocrine and neuronal markers after HIF-2 activation [78]. HIF-2 has also been shown to activate the expression of the transcription marker Oct-4 which is associated with stem cell pluripotency and de-differentiation [13]. However, juxtaglomerular renin-producing cells not only lose characteristic markers during the transformation into EPO-producing cells but also gain markers typical of the new phenotype. It should also be mentioned in this context that not every cell type can be transformed into an EPO-expressing cell by HIF-2α stabilization [87, 93, 94]. Even in close relatives of renin^+^ cells, like VSMCs which develop from renin-producing cells during nephrogenesis, EPO expression cannot be induced by HIF-2α stabilization [5, 100, 106]. Only after a prior transformation of VSMCs and EGM cells into renin^+^ cells due to genetic RAAS activation, these cells can be further transformed into EPO-producing cells [6].

Further aspects on EPO-expressing cells are reviewed in the article of Wenger et al. also published in this Special Issue on “Kidney Control of Homeostasis.”

## Renin cell plasticity: opportunities beyond renin production

The plasticity of renin^+^ cells is evident in the reversible recruitment of VSMCs and EGM cells during homeostatic challenges, in their ability to replace specialized intraglomerular cells and their ability to change their endocrine function by producing EPO instead of renin. This plasticity could offer a number of possibilities for therapeutic approaches in the future.

With regard to glomerular diseases, a treatment with RAS inhibitors has been shown to be beneficial in patients [32, 59, 110, 116]. Promising data from studies in mice indicate that juxtaglomerular renin-lineage cells could be used to replace intraglomerular mesangial cells or podocytes [53, 85, 105]. However, this repair mechanism seems to be insufficient in chronic progressive glomerular disease. A deeper understanding of the factors and signaling pathways that control this regeneration process will be necessary to specifically activate cells of the renin-lineage for glomerular repair.

With regard to the potential EPO-producing ability of renin-producing cells, one could speculate about new therapeutic opportunities to treat anemia in patients with chronic kidney disease. If the complex transformation mechanism could be unraveled, a targeted endocrine phenotype switch in juxtaglomerular renin cells could be induced for treatment. Previous findings suggest that the loss of renin production due to transformation of juxtaglomerular cells into EPO-producing cells may be compensated by recruitment of VSMCs, as observed in physiological stress situations [19, 48]. In this context, it will also be important to elucidate if the transformation of juxtaglomerular renin^+^ cells into EPO^+^ cells is reversible as observed for recruited VSMCs or EGM cells.

A possible transformation into EPO-producing cells should be considered, when evaluating the long-term effects of PHD inhibitor treatment on renin-producing cells. PHD inhibitors are a novel class of drugs that have recently been approved for the treatment of anemia in chronic kidney disease (Roxadustat is the first PHD inhibitor approved in the EU since September 2021) [12]. They interfere with the hypoxia signaling pathway and lead to the stabilization of hypoxia-inducible factors [34]. Therefore, it would be possible that EPO induction could occur in juxtaglomerular renin cells.

The existence of interstitial renin-expressing cells adds a new layer to the field of renin research. So far only few aspects are known about these cells. It appears that additional interstitial cells are recruited to express renin under certain conditions, for example, during anemia-induced hypotension or in experimental renal injury. Interestingly, these cells can also fulfill multiple endocrine functions simultaneously [6, 70].

## Challenges for future renin research

To this date, several typical markers and signaling pathways involved in conveying the identity of juxtaglomerular renin-producing cells and the recruitment of additional cells have been elucidated. However, less is known about the detailed regulatory mechanisms that govern the migration of renin cells into damaged glomeruli and their differentiation into another cell type. Similarly, little is known about the endocrine shift into EPO-producing cells. Therefore, it will be of future interest to unravel the specific pathways that govern the transformational processes and phenotypic changes of renin-expressing cells. This would allow the development of selective drugs to specifically manipulate these cells and influence their transformation.

Furthermore, it will be of interest to determine the function and relevance of interstitial renin expression. To this aim, the cells should be characterized in more detail to identify differences and similarities to juxtaglomerular cells in terms of their phenotype and regulation. One challenge will be to analyze the interstitial renin^+^ cells without simultaneously affecting the juxtaglomerular cells to determine whether the interstitial cells cooperate with the systemic RAAS or function independently.
